# The Molecular Mechanism of Opening the Helix Bundle Crossing (HBC) Gate of a Kir Channel

**DOI:** 10.1038/srep29399

**Published:** 2016-07-21

**Authors:** Xuan-Yu Meng, Shengtang Liu, Meng Cui, Ruhong Zhou, Diomedes E. Logothetis

**Affiliations:** 1 Institute of Quantitative Biology and Medicine, SRMP and RAD-X, Collaborative Innovation Center of Radiation Medicine of Jiangsu Higher Education Institutions, Soochow University, Suzhou 215123, China; 2 Department of Physiology and Biophysics, Virginia Commonwealth University, School of Medicine, Richmond, Virginia; 3 IBM Thomas J. Watson Research Center, Yorktown Heights, NY 10598, USA; 4 Department of Chemistry, Columbia University, New York, NY 10027, USA

## Abstract

Inwardly rectifying K^+^ (Kir) channels, serving as natural molecular nanomachines, transport potassium ions across the plasma membrane of the cell. Along the ion permeation pathway, three relatively narrow regions (the selectivity filter (SF), the inner helix bundle crossing (HBC), and the cytosolic G loop) may serve as gates to control ion permeation. Our previous molecular dynamics simulations based on the crystal structure of a Kir3.1 chimera revealed the possible gating mechanism of the G loop gate. Here, we introduced a proline mutation in the inner helix and obtained a channel model of the open HBC gate. The open HBC gate reaches 0.6 nm in diameter, which allows partial hydrated K^+^ ions to pass through. During the gating process, both the transmembrane helices TM1 and TM2 cooperatively rotate in a counterclockwise direction (viewed from the extracellular side) with the aid of the phospholipid PIP_2_. Only when all the transmembrane helices adopt a counterclockwise rotation, the HBC gate can be stabilized in the open state. We estimate that introduction of the proline mutation decreases the energy required to open the HBC gate by about 1.4 kcal/mol (ΔΔG).

Inwardly rectifying potassium (Kir) channels, as the structurally simplest branch of the superfamily of potassium-selective ion channels, are distinguished from other potassium channels by their biophysical property of inward rectification that allows more potassium ions to enter into the cell at membrane potentials below E_K_ than to leave the cell at membrane potentials above E_K_. Kir channels consist of fifteen members classified into seven subfamilies Kir1–7. They are found in many cell types and serve various physiological functions such as the regulation of heart rate, insulin release and membrane excitability. One prominent feature of Kir channels is their regulation by the signaling phospholipid phosphatidylinositol 4,5 bisphosphate (PIP_2_)^1^,^2^. PIP_2_ molecules bind to Kir channels through strong electrostatic interactions formed between the PIP_2_ phosphate groups and basic residues in the C-linker/Slide helix of the channels^3^. All Kir channels require PIP_2_ to maintain the normal physiological function and aberrant PIP_2_-Kir interaction (e.g. caused by specific mutations) may lead to channelopathies, such as the Bartter and Andersen syndromes^4^.

In 2007, the MacKinnon lab designed and crystallized a chimeric mammalian Kir3.1 channel in which the transmembrane domain was replaced by the corresponding region of the prokaryotic KirBac1.3 channel^5^. Subsequent functional reconstitution of this chimera by our group into planar lipid bilayers showed that it behaves like a typical Kir channel that requires PIP_2_ for activation and displays Mg^2+^-dependent inward rectification^6^. The structure of the Kir3.1chimera indicates two states of the cytosolic G-loop gate: the dilated (open) and constricted (closed) forms. The availability of these two structures enabled us using molecular dynamics (MD) simulations to demonstrate the molecular mechanism by which PIP_2_ mediates the opening of the G-loop gate; however, another gate in the transmembrane domain, known as the helix bundle crossing (HBC) gate, remained in the closed state throughout the simulations; prolongation of the simulation time also failed to capture this gate in the open state^7^. Three to four years following the elucidation of the Kir3.1 chimera structure, the MacKinnon lab further contributed a series of Kir2.2 and Kir3.2 channel structures^8^,^9^,^10^,^11^. Of special note is the complex structure of a mutant Kir3.2 channel bound to PIP_2_ which captured a half-open state of the HBC gate^10^. A fully open model was constructed based on the half-open structure^10^. However, MD simulations we performed on this fully open model showed that the HBC gate turned to a half-open state within a very short simulation time.

Several lines of experimental evidence have indicated that a highly conserved glycine residue in the middle of TM2 plays an important role in channel gating by introducing flexibility to the TM2^13^. Replacement of the residue right after this Gly by a proline in GIRKs stabilizes a kink and generates a constitutively active channel^13^,^14^.

We thus introduced this proline mutation at the same position in the Kir3.1 chimera, M170P, corresponding to S170P in GIRK1, in order to investigate the HBC gating process by using MD simulation methods. As aforementioned, both structures of the Kir3.1 chimera, the constricted and dilated forms, were considered. Thus we worked with four mutant systems: the M170P constricted form in the absence and presence of PIP_2_ and the M170P dilated form in the absence and presence of PIP_2_; together with four WT systems (constricted and dilated forms in the absence and presence of PIP_2_), totally eight comparable simulation systems were used in this study. Interestingly, among the eight simulations only in the M170P dilated holo system the HBC gate opened. Transition from the closed to the open state of the HBC gate involved both bending and rotating motions of the inner TM2 helices. A series of hydrophobic interactions within the transmembrane helices and the Slide helix changed during the opening transition. In this system we observed potassium ions traveling along the central permeation pathway, crossing the gates and finally entering the cytosol.

## Methods

### Molecular Models

The crystal structures of the Kir3.1 chimera (between the WT Kir3.1 and KirBac3.1) lacked the N-terminus (both dilated and constricted forms), the C-linker region (constricted form) as well as the side chains of some residues. Modeller v9.5^15^ was used to build these missing channel regions and the complete models were subjected to energy minimization using the CHARMM program with an implicit membrane/solvent Generalized Born (GB) model for 1000 steps of a steepest descent minimization^16^. Details of the model refinement process have been previously described^17^. The M170P mutation was introduced on the refined models by using Discovery Studio (Accelrys Inc., San Diego, CA, USA) software.

### System Setup and Molecular Dynamics Simulations

The M170P dilated/constricted structures were immersed in an explicit POPC bilayer of ~35 Å thickness and were solvated in 0.15 M KCl. The SPC water model was used. Each of the four mutant systems (M170P dilated holo, M170P dilated apo, M170P constricted holo and M170P constricted apo) was run for 100 ns MD simulations using the GROMACS v4.5.4 program^17^ with the GROMOS96 53a6 force field^18^. Long range electrostatics were calculated using the particle mesh Ewald (PME) method^19^ with 12 Å cut-off. Van der Waal interactions were modeled using Lennard-Jones 6–12 potentials with 14 Å cut-off. All simulations were conducted at a constant temperature of 300 K using the Berendsen thermostat. The system pressure was coupled at isotropic (X + Y, Z) directions referenced to 1 bar using the Berendsen method^20^. All bonds were constrained with the LINCS algorithm^21^. The time step was 2 fs and the neighboring list was updated every 10 time steps. The topology parameters for PIP_2_ were generated from the Prodrg server^22^ and atomic charges of PIP_2_ were used as in our previous work^23^. The lipid parameters were obtained from Tieleman’s website (http://moose.bio.ucalgary.ca).

Prior to production runs, energy minimization of 3000 steps of steepest descent was carried out on each system followed by a 0.5 ns two-step equilibration process. In the first 0.2 ns, channels, K^+^ ions and PIP_2_ were position-restrained using a constant force of 1000 kJ/mol/nm^2^, allowing lipids and water molecules to move freely. Then the constant force was reduced to 10 kJ/mol/nm^2^ in the following 0.3 ns equilibration. An electrical field of 0.06 V/nm was applied in this step as well as the production run, along the z-axis of the box to maintain the lower potentials in the intracellular side. The treatment of the electrical field has been detailed in previous work^24^,^25^. A 100 ns production run was conducted on each system and coordinates were saved every 10 ps for analysis. VMD^26^ and Chimera^27^ were used for visualization.

### Analysis of MD runs

The mutant systems reached equilibration after 10 ns simulations according to root mean square deviations (RMSD) of the channel Cα atoms (Fig. S1). The 10–100 ns trajectories of each system were used for analysis.

The Simulaid program^28^ was used to calculate the rotating and bending motion of transmembrane helices. The interactions of hydrophobic contacts were also calculated using the Simulaid program. The Simulaid outputs for interactions were reorganized with in-house scripts for facility of comparison among the systems. Principal Components Analysis (PCA) was conducted to extract the collective motions of the channel from the MD simulation trajectory. It describes the motions with a set of eigenvector and eigenvalue pairs, which are obtained by diagonalizing the covariance matrix of the Cα atomic positional fluctuations^29^,^30^. The analysis program g_anaeig within GROMACS was employed to conduct PCA and the first eigenvector describes the motion of TM2 which is associated with dilation of the HBC gate.

## Results

### TM2 Bending at G169

**Figure 1:**
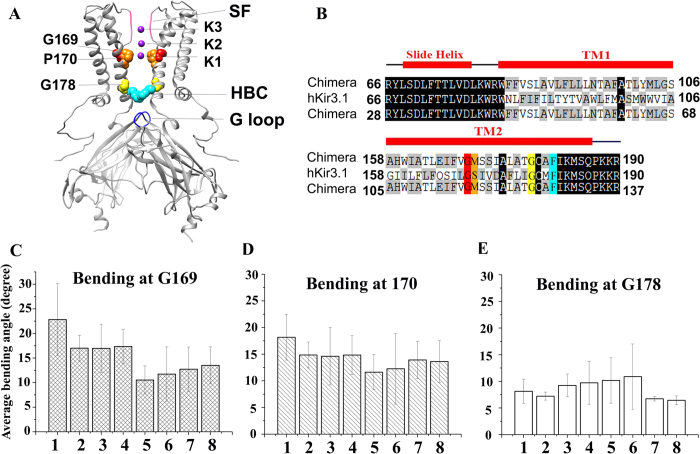
(**A**) Mutation of M170P is introduced in the Kir3.1 chimera. Several structural elements involved in this study are denoted: Selectivity Filter (SF, in pink), Helix Bundle Crossing gate (HBC, in cyan), G loop gate (in blue); G169, M170P and G178 are shown with red, orange and yellow VDW spheres, respectively; three potassium ions K1, K2 and K3 which passed through all the gates in 100 ns simulations. (**B**) Sequence alignment between Kir3.1 Chimera and hKir3.1. We used the hKir3.1 residue number for discussion, and the original residue number of the chimera was also listed. (**C**–**E**) Average bending degree of the TM2 at kink points G169 (**C**), 170 (**D**) and G178 (**E**) over 10–100 ns MD simulations. Systems 1–8 represent M170P dilated holo, M170P dilated apo, M170P constricted holo, M170P constricted apo, WT dilated holo, WT dilated apo, WT constricted holo and WT constricted apo, respectively.

A proline kink at position 176 in GIRK4^*^ enhances channel gating by increasing the open probability of the channel^13^. A similar behavior was also observed in the heteromeric GIRK1(S170P)/GIRK4(S176P) channel^14^. We introduced the proline mutation at the same position in the Kir3.1 chimera, M170P, to test whether the channel could be opened with the aid of the mutation (Fig. 1A,B). The average bending degree was calculated based on 100 ns MD simulations. With a proline mutant introduced at position 170, the TM2 helix bent significantly at position G169. The relevant bending degrees in all mutant systems were larger than in the WT systems. The M170P dilated holo system showed the largest average bending degree around 23° at G169 and 18° at M170P (Fig. 1C,D). These effects were in contrast to the bending degree seen at G178, another glycine in the TM2 which is also capable of introducing flexibility to the helix. While all the systems displayed a slight bending at G178, no significant differences were found between the WT and mutant systems (Fig. 1E). Therefore, the main bending of the TM2, as expected, arose from the proline kink at 170.

### Potassium ions passing the channel

**Figure 2:**
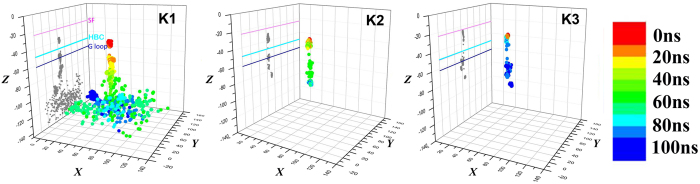
Distribution of passing K ions over simulation time in the M170P dilated holo system. Coordinates of each K ion were plotted and color-coded according to the simulation times. Grey dots represented the projection of the ion coordinates on the Y–Z plane. The positions of the SF, HBC and G loop gate along the channel axis were also denoted with pink, cyan and blue lines, respectively.

At the beginning of the simulation, three potassium ions K1, K2 and K3 were positioned in the central pathway where their coordinates were directly copied from the crystal structure of the Kir3.1 chimera (Fig. 1A). The water molecules co-crystalized with K ions were also reserved in the simulations models. The motion of each ion was monitored in each system as a function of the simulation time. It was only in the M170P dilated holo system that we observed the three K ions moving along the central pathway and crossing the HBC gate. The moving traces of the three ions were color-coded according to the simulation time (Fig. 2). K1 crossed the HBC gate at 10.8 ns and then lingered at the G loop gate until 26.6 ns. During its travel to the cytosolic side, K1 was sequentially trapped by cytoplasmic-pore-lining residues E300 and D260 and finally entered into the soluble cytosolic phase at around 50.3 ns. The K2 and K3 ions lingered for 60 ps (50.87 ns to ~50.93 ns) and 80 ps (82.24 ns to ~82.32 ns) at the HBC gate, respectively and then passed through the G loop gate at 53.91 ns and 84.54 ns, respectively. They were trapped by D260 residue of different subunits at the end of the 100 ns simulations.

A recent study from Köpfer *et al*. suggested that K ion permeation obeys a ‘knock-on’ mechanism that direct coulomb repulsion is the key to high-efficiency conductance of K channels^31^. In our simulations, we set water molecules occupying the S2 and S4 positions in the SF at the beginning of the simulation; water molecules co-crystalized in the cavity were also reserved. The three K ions were separated by water molecules in the initial setup, consistent with the mechanism of co-translocation of ions with water^32^,^33^,^34^,^35^,^36^. In this case, it took around 40 ns for one K ion to pass through the HBC gate in the MD simulations, corresponding to a 4 pA current, which approaches the current magnitude of S176P Kir3.4^*^ based on single channel recordings^13^.

**Figure 3:**
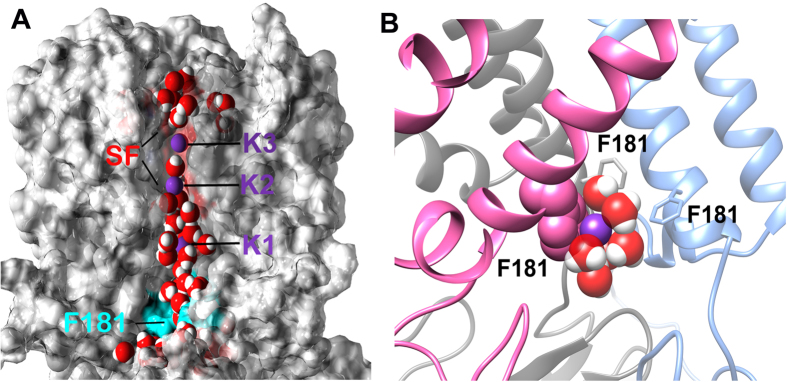
(**A**) Water penetrated the HBC gate prior to the passing of K ions. This snapshot was captured right before the first K ion (K1) passed the HBC gate. The SF and HBC gate (F181) were highlighted in the red and cyan surface, respectively. The HBC gate stabilized at the open conformation and allowed water molecules to pass through. K1 was coordinated by water molecules in the inner cavity. (**B**) A snapshot of K3 passing the HBC gate. K3 was partially hydrated when it passed the HBC gate and simultaneously a cation-π interaction formed between K3 and F181 in the subunit A. The front subunit was removed on both panels to reveal the permeation pathway.

The HBC gate is comprised of bulky hydrophobic residues (Phe, Met, Leu or Val) in Kir channels. Our simulations showed that the HBC gate in the closed state occludes water molecules, while the open state of the gate allows water to pass (Fig. 3A). Our simulations also indicated that, when a potassium ion passed through the gate, the ion formed cation-π interactions with one of the F181 residues in the gate on one side, while it was surrounded by several water molecules on the other side (Fig. 3B). This configuration delayed the ion at the HBC gate for about 80 ps.

### Dilation of the HBC gate

**Figure 4:**
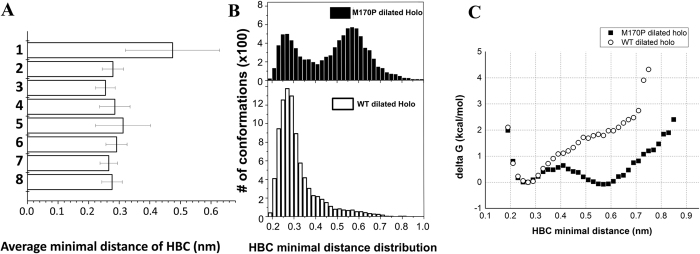
(**A**) Average minimal distance of HBC gate over simulation time in eight simulation systems. (**B**) Frequency count of the HBC minimal distance over 10–100 ns MD trajectories in the M170P dilated holo and WT dilated holo systems, respectively. Data of minimal distances were counted in increments of 0.02 nm. (**C**) Free energy changes of HBC gate opening was estimated using 
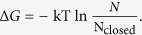

We monitored the minimal distance of two Phe residues (F181) between opposite subunits and took the shortest distance from the pair as the minimal distance of the HBC gate. The average minimal distance of the HBC gate throughout the simulation time for each of the 8 systems is summarized in Fig. 4A. The M170P dilated holo showed the largest minimal distance at 0.48 nm (system 1). The rest of the systems were generally around 0.3 nm wide at the HBC gate. To further characterize the gate, we counted the occurring frequency of the minimal distance in increments of 0.02 nm using the data from the M170P dilated holo system and the WT dilated holo system as representatives (Fig. 4B). The frequency of the minimal distance in the M170P dilated holo system showed two clear peaks: the first peak was located between 0.24–0.30 nm and the second between 0.54–0.60 nm; while the WT dilated holo system displayed only one peak, which resembled the first peak in the former mutant system. The two peaks perfectly correspond to the closed and open states of the gate, respectively. This result can be used as direct evidence to quantitatively distinguish the open from the closed state of the gate.

We further used this frequency count to estimate the free energy changes (ΔG) for opening the HBC gate (Fig. 4C). In this calculation, the most stable closed conformations in each system (HBC minimal distance ~0.26 nm, Fig. 4B) were defined as the reference conformations and their free energies were set to 0 kcal/mol. Relative to this, we estimated the free energies of the rest of the conformations. Fig. 4C clearly shows that, the M170P dilated holo system has two minima at ~0.26 nm and 0.55–0.6 nm, corresponding to the closed and open conformations, respectively. The free energy of the open state is −0.08 kcal/mol compared to the closed one; the energy barrier between the minima is about 0.6 kcal/mol. In contrast, the WT dilated holo system shows one minimum at 0.26 nm corresponding to the closed state. Its free energy increases through opening of the HBC gate. By comparing the two systems, the M170P mutation decreases about 1.4 kcal/mol (the free energy barriers are 0.6 and 2 kcal/mol for the M170P and WT channels, respectively) for opening the HBC gate (0.55–0.6 nm).

### Rotation of Transmembrane Helices

In addition to the bending motion of the TMs, the opening of the HBC gate is associated with a unique rotational motion of the helices. As shown on Table 1, all the TMs in the M170P dilated holo system showed a counterclockwise rotation. In contrast, in the rest of seven systems at least one helix underwent a clockwise rotation. Thus, the counterclockwise rotation of all TM2 helices might be a necessary condition for the opening of the HBC gate.

Another regular pattern seen in this table is that, the dilated systems (both the mutant and WT) possess more counterclockwise-rotating helices compared to the constricted systems, implying a correlation between the G loop gate and the HBC gate. The G loop gate being in the dilated conformation benefited the counterclockwise rotation of the helices. The two gates should open in a sequential manner such that dilation of the G loop gate is a prerequisite to the opening of the HBC gate.

**Figure 5:**
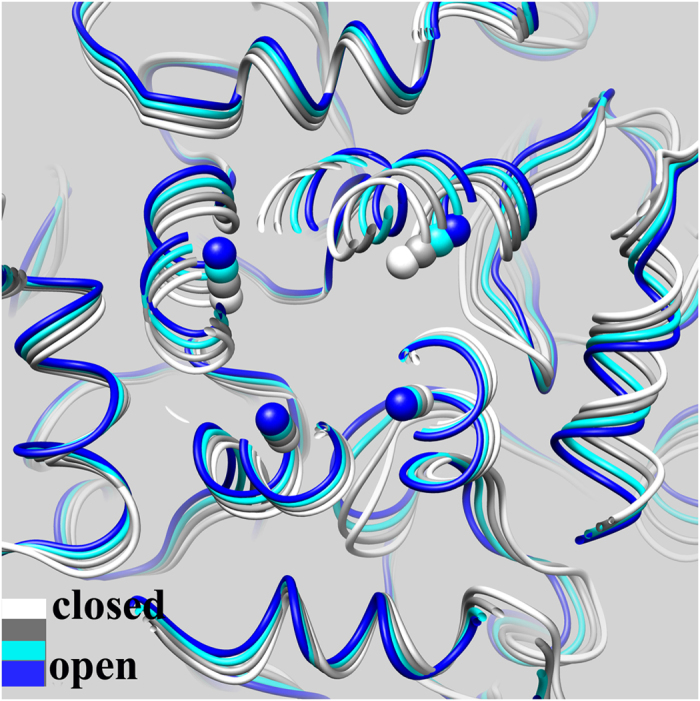
The first eigenvector from PCA analysis on trajectory of 10–100 ns of the M170P dilated holo system. The Cα atoms of HBC residues are shown as spheres. Transitions from white to blue indicated the counterclockwise rotation of TM2 residues associated with the opening of the HBC gate. Thirty frames were generated to describe the collective motion of the eigenvector using the Gromacs inset program g_anaeig and four of them were selected to show for visual clarity.

We also conducted a Principal Components analysis (PCA) on the M170P dilated holo system (Fig. 5). The PCA results visualized the counterclockwise rotation of the TM2 helix which agreed well with the rotational degree calculation. Such counterclockwise rotation of the TM2 caused an outward motion of F181 and contributed to the dilation of the HBC gate.

### Residue Interactions Involving the HBC Gate Opening

As mentioned earlier, opening of the HBC gate was attributed to the bending motion of TM2 at the hinge G169 and the rotating motion of both the TM1 and TM2 helices in a counterclockwise direction (viewed from the outside). These conformational changes must be accompanied by changes in residue interactions. We therefore monitored hydrophobic interactions within the WT and M170P dilated holo systems. Percentages of hydrophobic interactions (survival percentage values for a given interaction) were calculated and used to make comparisons between the two systems (see Table S2). The results and discussion are based on total percentages, which sum the interaction percentages from all four subunits in order to simplify the comparison and make it clear.

**Figure 6:**
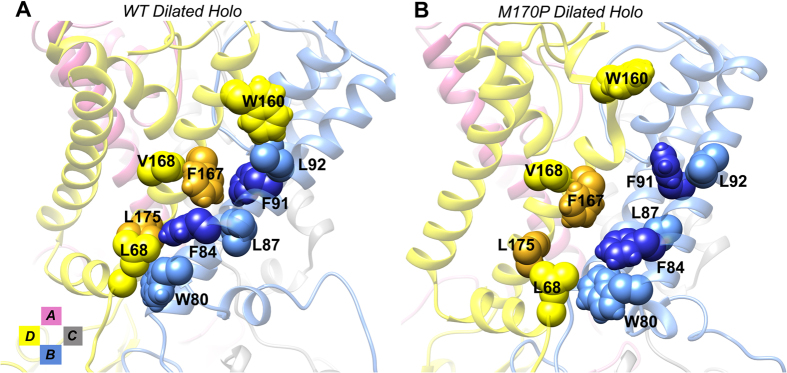
Strong inter-subunit hydrophobic interactions formed between TM2/SLH and TM1 in the WT dilated holo (A) but disrupted in the M170P dilated holo (B).

In the WT dilated holo system, the TM1 stabilized the TM2 of an adjacent subunit by a group of hydrophobic contacts. As seen in Fig. 6A and Table S2, F84 in the TM1 formed contacts with V168 and L175 in 73% and 63% of simulation time, respectively; these contacts weakened and the percentages of interactions decreased to 30% and 48%, respectively, in the M170P dilated holo system. A similar decreased pattern was found in the pairs L87-F167, F91-F167, L92-W160 and L92-F167. The overall hydrophobic interaction between TM1 and TM2 from adjacent subunits was weakened during the transition from the closed to the open HBC gate (percentage 7.21 in M170P dilated holo vs. 11.53 in WT dilated holo). In addition to hydrophobic residues, we also calculated the percentage of all TMs residue contacts. The results showed the same decreasing trend in the mutant dilated holo compared to the WT system (percentage 23.90 vs. 30.12). This result suggests that the transition from the closed to the open HBC gate is associated with the slight dissociation between the TM1 and TM2 helices of adjacent subunits. It is difficult to tell whether the dissociation arises from the counterclockwise rotation of the helices or the dissociation occurs firstly to benefit the rotation. However, it is clear that opening of the HBC gate requires disruption of specific contacts between the TM1 and TM2 helices that contribute to the gating energy barrier.

**Figure 7:**
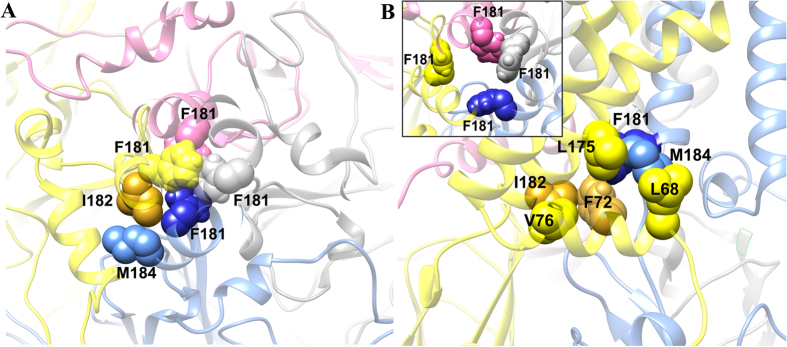
(**A**) Hydrophobic interaction network in the WT dilated holo system to stabilize the closed state of the HBC, viewed from the extracellular side. (**B**) Hydrophobic interaction network in the M170P dilated holo system to stabilize the open state of the HBC, a side view; inserted panel is a top-down view to show the open HBC gate.

The HBC gate consists of four Phe residues (F181) in the Kir3.1 chimera. In most cases of the WT MD trajectories in which the HBC gate remained closed, π-π interactions were formed between one pair of diagonal F181 residues (F181 in yellow and silver in Fig. 7A); the other two usually packed around the pair and strengthened the hydrophobicity of the gate. The ion pathway was completely blocked by the compact contacts. Neither ions nor water molecules could cross the gate in the simulations. Additionally, as seen in Fig. 7A, the F181 in blue formed hydrophobic interactions with the orange I182 from the adjacent yellow subunit; and the orange I182 also contacted the M184 in the blue subunit. The I182 and M184 stabilized the closed state of the gate by forming hydrophobic interactions with the F181 residues.

When the HBC gate transitioned to the open state in the mutant dilated holo system, as can be seen in the top left panel of Fig. 7B, the four F181 residues were kept separated from each other to make room for the passing of the water and ions. In this circumstance, the F181 residues formed new hydrophobic interactions with surrounding residues to stabilize themselves. To illustrate the new hydrophobic network, we still used the interactions formed within the yellow and blue subunits as representatives. As seen in Fig. 7B, the blue F181 residue formed hydrophobic interactions with the F72 and L175 residues in the yellow subunit. The blue M184, which stabilized the F181 in the closed state, formed contacts with L68 and L175 in the yellow subunit. The orange I182 that was stabilizing the F181 in the closed state, turned to form hydrophobic interactions with the F72 and V76 in the yellow subunit. The L68, F72 and V76 in the Slide Helix and the L175, F181 and M184 in the TM2 formed a hydrophobic core to stabilize the open state of the HBC gate. In other words, the Slide Helix plays a critical role in stabilizing the TM2 helix in the open state of the channel.

### Roles of PIP_2_ for the HBC gate opening

PIP_2_ as one of the most important co-factors for Kir channel activity, involved in the opening of both gates. Our previous simulations indicated that PIP_2_ drives the movements of the N-terminus and C-linker, leading to a cascading effect involving stabilization of the CD-loop to an active conformation and weakening of the R313-E304 salt-bridge that restrains the G-loop gate^7^. In the process of HBC gate opening, PIP_2_ molecules are stabilized at their binding site in the interface of transmembrane and cytosolic during 100 ns long simulations. The binding mode of PIP_2_ with the channel does not change and the conserved PIP_2_-channel salt bridges remain unchanged between the M170P dilated and WT dilated systems (Fig. S3). However, PIP_2_ enhanced the bending of TM2 in the open HBC gate. The presence of PIP_2_ caused a 7° greater bending of TM2 at G169 (Fig. 1C, M170P dilated holo vs. M170P dilated apo). Moreover, with PIP_2_, the M170P dilated channel displayed the counterclockwise rotation of all eight TM helices (Table 1). It is still not very clear how the PIP_2_ interactions lead to the opening of HBC gate. However, our present study suggests that not only the G-loop gate but also the opening of the HBC gate relies on the presence of PIP_2_.

## Discussion and Conclusion

Our previous work illustrated the molecular mechanism of opening the intracellular G-loop gate of a Kir3.1 chimera^7^. Yet opening of the HBC gate could not be captured by our simulations in that study. With the aid of a proline mutation at position 170, we successfully simulated the opening of the HBC gate and observed potassium ions passing through the channel. The proline mutation decreased the free energy of opening of the HBC gate by about 1.4 kcal/mol. This result was consistent with the experimental observation that a relevant proline mutation dramatically increased the open probability of heteromeric GIRK1/GIRK4^14^ and GIRK4*^13^ channels.

Proline mutations increased the bending of the TM2 helices in all four systems (constricted/dilated apo/holo). However, only the dilated holo system with M170P achieved an open HBC gate, indicating that PIP_2_ and opening of the G-loop gate are two critical prerequisites for the opening of the HBC gate. Currently available crystal structures also suggest that the G-loop gate opens prior to the HBC gate^10^,^37^,^38^. In addition to the half opened Kir3.2 structure, the other apparent open structure is based on KirBac3.1 with the S129R mutation (PDB 3ZRS). The open HBC gate was captured by utilizing the electrostatic repulsion of Arg residues to achieve the bending and rotation of the TM2 helix. The TM2 bends 20° at G120 (corresponding to G169 in Kir3.1 chimera) and rotates counterclockwise about 25° (viewed from the extracellular side)^37^. It is in agreement with our calculations that the M170P mutation causes a 23° bending and up to a 12°counterclockwise rotation. It is not surprising that S129R leads to a stronger rotation of TM2 under the electrostatic effects. Another MD simulation study published in 2015 based on KirBac1.1 used a similar strategy. They introduced a Glu at the same position and observed the opening of the lower part of the TM2 helix. However, their simulation indicated that the HBC gate opened earlier than the twisting motion of the cytoplasmic domain (related to the G-loop gate), which might indicate subtle variations between different channels^39^.

Our detailed analysis of the minimal distance of HBC gate showed us that the gate stabilized at 0.24–0.30 nm or 0.54–0.60 nm in most cases, which corresponded to the closed and open states of the HBC gate, respectively. Here the minimum distance calculation is based on the center of mass of atoms. If we consider van der Waals (vdW) radii, the distance for the two states would be roughly estimated as 0.06–0.12 nm and 0.36–0.42 nm, respectively (deducting the radii of two carbon atoms that may constitute two ends of the minimal distance of HBC gate - in fact the hydrogen atoms of F181 also constituted the ends of the minimal distance in some conformations. In that case the minimal distance would be even larger than our estimated values). On the other hand, hydrated K ions are 0.396 nm in radius. The HBC gate with 0.36–0.42 nm in width just allows ‘half’ hydrated K ion to pass, which implies that half of the K ion is hydrated and the other half is exposed to the F181 residue. As we observed in the MD simulation, the exposed half facing the F181 residue forms cation-π interactions with the benzene ring of F181 (Fig. 3B).

## Additional Information

**How to cite this article**: Meng, X.-Y. *et al*. The Molecular Mechanism of Opening the Helix Bundle Crossing (HBC) Gate of a Kir Channel. *Sci. Rep.* **6**, 29399; doi: 10.1038/srep29399 (2016).

## Supplementary Material

Supplementary Information

## Figures and Tables

**Table 1:**
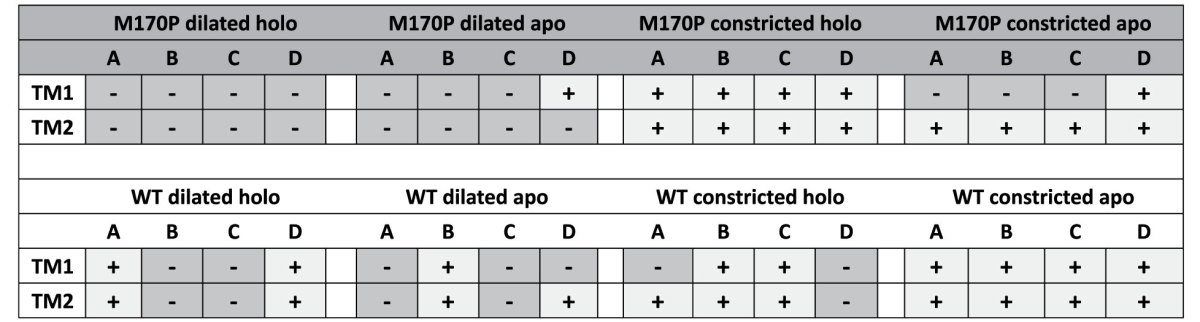
The clockwise (+)/counterclockwise (−) rotation of the transmembrane helices.

The average rotational degrees of TM1 and TM2 in each subunit were calculated over 10−100 ns MD simulations. Along the axis from extracellular to intracellular side (viewed from the outside to the inside membrane), the plus (+, in light grey) indicated a clockwise rotation of TM, while a minus (−, in dark grey) indicated a counterclockwise rotation. The magnitudes of the degrees of rotation are listed on Table S1.
