# Supplementary material for: The Molecular Mechanism of Opening the Helix Bundle Crossing (HBC) Gate of a Kir Channel
Source: Sci Rep. 2016 Jul 21;6:29399. doi: 10.1038/srep29399 (PMC4954981; doi:10.1038/srep29399)
Supplement: Supplementary Information [file srep29399-s1.pdf]

The Molecular Mechanism of Opening the Helix Bundle Crossing (HBC) Gate of a Kir Channel

Xuan-Yu Meng[1,2], Shengtang Liu[1],Meng Cui[2], Ruhong Zhou[1,3,4], Diomedes, E. Logothetis[2*]

[1]*Institute of Quantitative Biology and Medicine, SRMP and RAD-X, Collaborative Innovation Center of Radiation Medicine of Jiangsu Higher Education Institutions, Soochow University, Suzhou 215123, China*

[2]*Department of Physiology and Biophysics, Virginia Commonwealth University, School of Medicine, Richmond, Virginia.*

[3]*IBM Thomas J. Watson Research Center, Yorktown Heights, NY 10598, USA*

[4]*Department of Chemistry, Columbia University, New York, NY 10027, USA*

Supplementary Materials

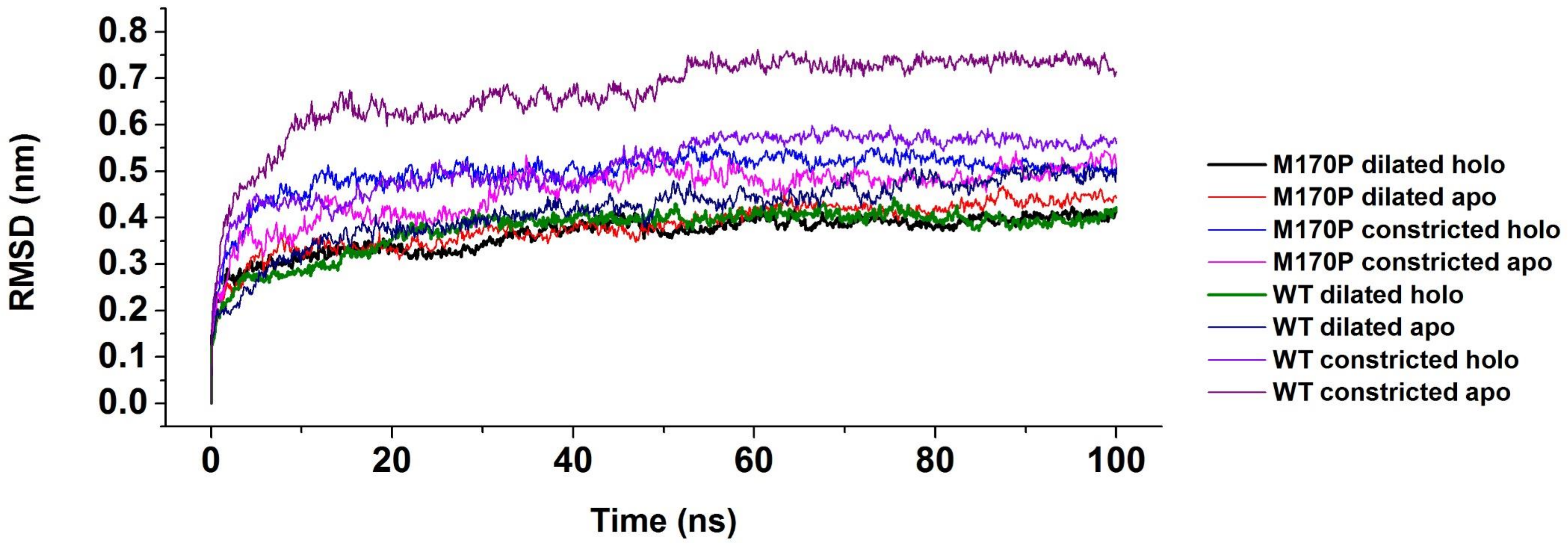

Figure S1. Root mean square deviation (RMSD) of the eight simulation systems involved in the study. RMSD was calculated based on the channel C-alpha atoms. All systems reached equilibrium after 10ns simulation according to the RMSD.

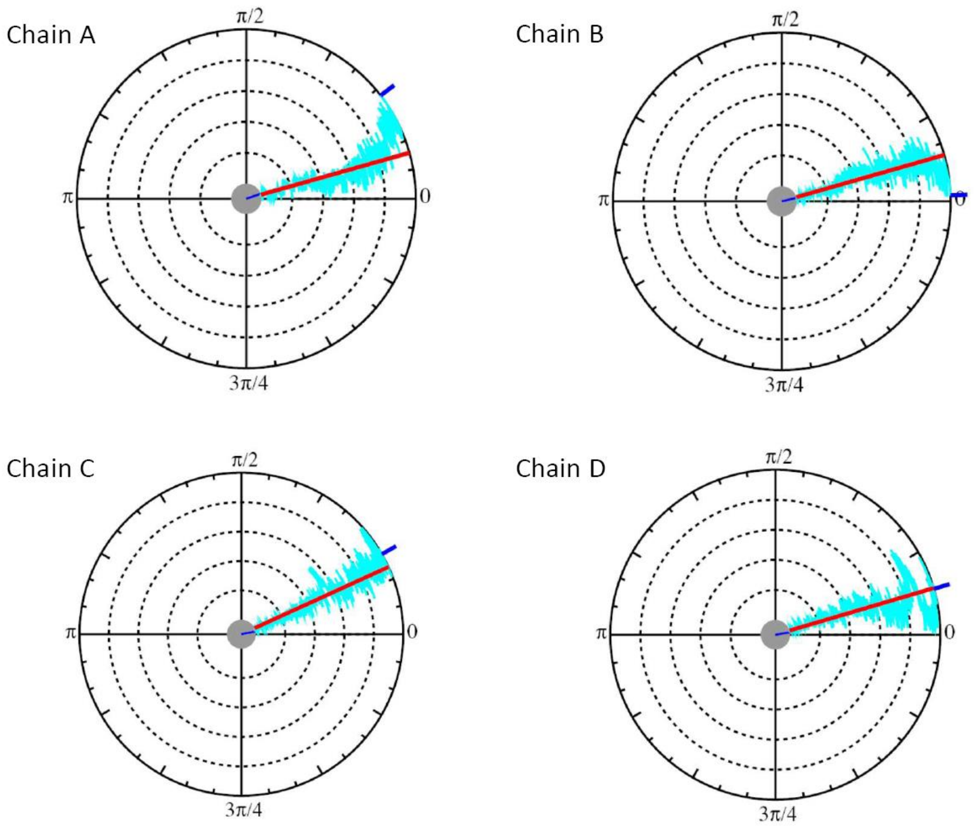

Figure S2. TM2 bending evolution upon 100ns simulation time in M170P dilated holo system (related to **Figure 1D**). The TM2 mainly bends at the P170 position. The bending degree of TM2 at P170 in each conformation was calculated and displayed by the cyan line. The short blue lines indicate the initial bending value (in the center) and the final (on the arc), respectively. The average bending degree is shown as a red line.

Table S1. Average rotational degrees of TM1 and TM2 in each subunit over 10-100ns MD simulations (related to Table 1).

	**M170P dilated holo**				**M170P dilated apo**				**M170P constricted holo**				**M170P constricted apo**			
	**A**	**B**	**C**	**D**	**A**	**B**	**C**	**D**	**A**	**B**	**C**	**D**	**A**	**B**	**C**	**D**
**TM1**	-6.21	-15.68	-2.12	-5.99	-3.13	-2.07	-7.46	3.66	2.88	0.5	6.12	0.43	-0.7	-6.99	-3.02	0.26
**TM2**	-2.83	-2.12	-1.13	-12.42	-7.4	-7.35	-0.29	-4.3	10.47	7.21	10.52	0.51	7.29	2.1	1.62	2.75

	**WT dilated holo**				**WT dilated apo**				**WT constricted holo**				**WT constricted apo**			
	**A**	**B**	**C**	**D**	**A**	**B**	**C**	**D**	**A**	**B**	**C**	**D**	**A**	**B**	**C**	**D**
**TM1**	4.88	-15.35	-5.53	5.34	-1.63	3.69	-4.38	-5.5	-0.44	0.4	3.25	-6.08	9.21	6.5	9.4	7.61
**TM2**	1.52	-20.64	-5.68	4.6	-2.09	7.95	-5.91	0.36	11.88	3.76	5.48	-1.12	13.44	9.74	25.7	17.28

Table S2. Percentage of residue hydrophobic interactions formed during 10-100ns simulation time (related to Fig. 6-7)

M170P dilated holo													WT dilated holo								
A	B	C	D	AC	BC	BD	AD	Total					Total	A	B	C	D	AC	BC	BD	AD
0	0	0	0	0.26	0.05	0.09	0	0.4	**L**	**68**	**W**	**80**	1.39	0	0	0	0	0.42	0.48	0.09	0.4
0	0	0	0	0.01	0.05	0.29	0.13	0.48	**L**	**68**	**M**	**184**	0.32	0	0	0	0	0.17	0.14	0.01	0
0	0	0	0	0	0	0.11	0.08	0.19	**F**	**72**	**F**	**181**	0.07	0	0	0	0	0	0.02	0.01	0.04
0.23	0.3	0.18	0.2	0	0	0	0	0.91	**F**	**72**	**I**	**182**	0.78	0.14	0.08	0.26	0.16	0	0	0.14	0
0.12	0.24	0.26	0.31	0	0	0	0	0.93	**V**	**76**	**I**	**182**	0.63	0.25	0.01	0.05	0.32	0	0	0	0
0.23	0.24	0.23	0.13	0	0	0	0	0.83	**F**	**84**	**W**	**80**	0.49	0.08	0.19	0.06	0.16	0	0	0	0
0	0	0	0	0.01	0.01	0	0.01	0.03	**F**	**84**	**V**	**168**	0.73	0	0	0	0	0.17	0.15	0.4	0.01
0	0	0	0	0.26	0.2	0.01	0.01	0.48	**F**	**84**	**L**	**175**	0.63	0	0	0	0	0.2	0.16	0	0.27
0	0	0	0	0.13	0.1	0.03	0.04	0.3	**L**	**87**	**F**	**167**	0.55	0	0	0	0	0	0.01	0.34	0.2
0	0	0	0	0.02	0	0	0	0.02	**L**	**87**	**P/M**	**170**	0.18	0	0	0	0	0.03	0.09	0.02	0.04
0	0	0	0	0.12	0.18	0.02	0.21	0.53	**F**	**91**	**F**	**167**	0.88	0	0	0	0	0.31	0.13	0.37	0.07
0	0	0	0	0.09	0.01	0	0.01	0.11	**F**	**91**	**P/M**	**170**	0.87	0	0	0	0	0.26	0.43	0.01	0.17
0	0	0	0	0	0.02	0	0	0.02	**L**	**92**	**W**	**160**	0.42	0	0	0	0	0.08	0.04	0.05	0.25
0	0	0	0	0.01	0.12	0	0.05	0.18	**L**	**92**	**F**	**167**	0.55	0	0	0	0	0.24	0.31	0	0
0	0	0	0	0	0	0.06	0.1	0.16	**L**	**175**	**F**	**181**	0	0	0	0	0	0	0	0	0
0	0	0	0	0.33	0.13	0.23	0.09	0.78	**L**	**175**	**M**	**184**	0.23	0	0	0	0	0.15	0.03	0.04	0.01
0	0	0	0	0.22	0.15	0.07	0.11	0.55	**F**	**181**	**F**	**181**	0.8	0	0	0	0	0.41	0.06	0.11	0.22
0	0	0	0	0.15	0.03	0.03	0.16	0.37	**F**	**181**	**I**	**182**	0.43	0	0	0	0	0.03	0.14	0.06	0.2
0	0	0	0	0.26	0.14	0	0.07	0.47	**I**	**182**	**M**	**184**	0.71	0	0	0	0	0.32	0.01	0.04	0.34

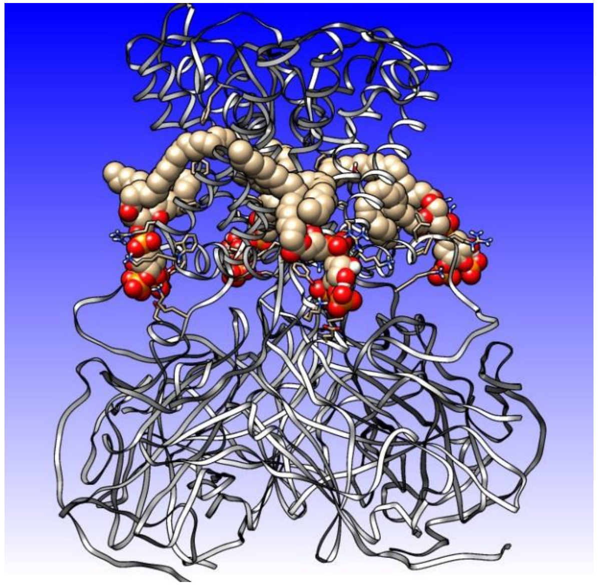

Fig. S3. PIP_2 binding site on the Kir3.1 chimera

Table S3. Residues that form salt bridges with PIP_2. Survival percentages of salt-bridge interactions were calculated from 10-100-ns-long MD simulations.

	WT Constricted				WT Dilated				M170P Dilated			
	A	B	C	D	A	B	C	D	A	B	C	D
K49				12								
R52		3	41	18					1		1	1
R66(inter)	1		1		18	46		14	1	18	22	34
K79	84	48	75	65	66	44	59	4	43	35	58	32
R81	3								13	25	5	7
K183	4				33	8	38	55		75	11	12
K188	26		1	52	44	68	85	68				83
K189	75		86	66	54	91	69	75	30	2	47	44
R190	29	3	4	40								1
R219					9	9	9	1				
